# Epidemiology and outcomes of choledocholithiasis and cholangitis in the United States: trends and urban-rural variations

**DOI:** 10.1186/s12876-023-02868-3

**Published:** 2023-07-27

**Authors:** Suqing Li, Leonardo Guizzetti, Christopher Ma, Abdel Aziz Shaheen, Elijah Dixon, Chad Ball, Sachin Wani, Nauzer Forbes

**Affiliations:** 1 Division of Gastroenterology and Hepatology, Department of Medicine, University of Calgary, TRW 6D62, 3280 Hospital Drive NW, Calgary, AB T2N 4Z6 Canada; 2 Alimentiv, Inc, London, ON Canada; 3 Department of Community Health Sciences, University of Calgary, Calgary, AB Canada; 4 Department of Surgery, University of Calgary, Calgary, AB Canada; 5 Division of Gastroenterology and Hepatology, University of Colorado Anschutz Medical Campus, Aurora, CO USA

**Keywords:** Choledocholithiasis, Cholangitis, Endoscopic retrograde cholangiopancreatography, Mortality, Epidemiology

## Abstract

**Background:**

Gallstone disease poses a significant health burden in the United States. Choledocholithiasis and cholangitis are common complications of gallstone disease for which data on current epidemiological trends are lacking. We aimed to evaluate temporal changes in hospitalization, management, and outcomes for patients with choledocholithiasis and cholangitis.

**Methods:**

The National Inpatient Sample was used to identify discharges for choledocholithiasis and cholangitis between 2005 and 2014. Temporal trends were evaluated via annual percent changes (APCs). Joinpoint regression was used to assess inflection points. Multivariable regression models were used to evaluate associations of interest.

**Results:**

From 189,362 unweighted discharges for choledocholithiasis and/or cholangitis, there was an increase in discharges for choledocholithiasis (APC 2.3%, 95% confidence intervals, CI, 1.9–2.7%) and cholangitis (APC 1.5%, 95% CI 0.7–2.2%). Procedural interventions were more likely at urban hospitals for choledocholithiasis (adjusted odds ratio, aOR, 2.94, 95% CI 2.72 to 3.17) and cholangitis (aOR 2.97, 95% CI 2.50 to 3.54). In-hospital mortality significantly decreased annually for choledocholithiasis (aOR 0.90, 95% CI 0.88 to 0.93) and cholangitis (aOR 0.93, 95% CI 0.89 to 0.97). In-hospital mortality between rural and urban centers was comparable for choledocholithiasis (aOR 1.16, 95% CI 0.89 to 1.52) and cholangitis (aOR 1.12, 95% CI 0.72 to 1.72).

**Conclusions:**

Hospitalizations for choledocholithiasis and cholangitis have increased between 2005 and 2014, reflecting a growing burden of gallstone disease. Hospital mortality between urban and rural centers is similar, however urban centers have a higher rate of procedural interventions suggesting limitations to accessing procedural interventions at rural centers.

**Supplementary Information:**

The online version contains supplementary material available at 10.1186/s12876-023-02868-3.

## Introduction

Choledocholithiasis is a common cause of extrahepatic biliary obstruction and the most frequent cause of cholangitis [1, 2]. Gallstone disease is rising in prevalence, representing the second most common principal admission diagnosis across all gastrointestinal, liver, and pancreatic conditions in United States [3, 4]. Amongst patients with cholelithiasis, 10–20% are found to have concomitant choledocholithiasis [5, 6].

Choledocholithiasis and cholangitis can result in significant morbidity and mortality and there have been significant updates in management approaches over the past decades [2, 7]. Given the less invasive nature and effectiveness of endoscopy compared to traditional open surgical common bile duct exploration (CBDE), endoscopic retrograde cholangiopancreatography (ERCP) has long been considered the first-line modality in the management of choledocholithiasis and cholangitis [7, 8]. However, with the advent of minimally invasive laparoscopic surgical techniques, there is debate as to whether ERCP or laparoscopic common bile duct exploration (L-CBDE) is the optimal first-line intervention, particularly in patients with an intact gallbladder [9, 10]. Coinciding with this, there have also been significant advances in endoscopic techniques and modalities in recent years (i.e. single-operator cholangioscopy with lithotripsy) which have allowed even difficult common bile duct stones to be managed endoscopically [11]. Several updates on the optimal management guidelines for cholangitis have also occurred throughout the past decade including the preferred method of initial biliary decompression and timing of biliary drainage [7, 12]. Given these factors, epidemiological data on incidence trends, admission patterns, management approaches, and outcomes for choledocholithiasis and cholangitis in recent years are needed but currently lacking.

In addition, with changing patient demographics and the use of advanced endoscopic and surgical procedures that require specialized training and resources often available only in tertiary care centers, the impact of residing in rural regions on clinical outcomes of patients with choledocholithiasis and cholangitis is unclear and has not been comprehensively evaluated. Several older studies have demonstrated urban-rural variability in access to ERCP and surgical techniques in the management of choledocholithiasis and cholangitis [13–15].

Therefore, we aimed to evaluate national temporal trends in hospitalization, management, post-procedural mortality, and post-procedural adverse events in the US for patients with choledocholithiasis and cholangitis, stratified by rural and urban hospital regions. Secondarily, we aimed to evaluate independent predictors of in-hospital mortality in patients with choledocholithiasis and cholangitis.

## Methods

### Study design and data source

We performed a retrospective study of data from the National Inpatient Sample (NIS) from 2005 to 2014. Data elements contained within the NIS include diagnoses and procedures coded using the *International Classification of Diseases, Ninth Revision, Clinical Modification* (ICD-9-CM), patient demographics, hospital characteristics, and measures of healthcare resource utilization.

### Study population

Our targeted study population was adult patients (≥ 18 years old) discharged from the hospital with a primary diagnosis of choledocholithiasis or cholangitis. Classifications of disease were based on ICD-9 CM codes (Supplementary Table 1). Admissions for choledocholithiasis were further subdivided by excluding secondary diagnostic codes for cholangitis in order to perform a sensitivity analysis of cases of choledocholithiasis alone. In the cholangitis cohort, admissions for cholangitis of all causes were included in the analyses. Admissions with any secondary or subsequent diagnosis code(s) indicating the presence of malignancy (Supplementary Table 2) were excluded from the study population.

### Outcomes and covariables

Our primary outcome measure was in-hospital mortality. Secondary outcomes included the use of surgical, radiologic, and endoscopic interventions and subsequent adverse events (AEs) including post-procedural mortality. Procedures were identified by the presence of one or more ICD-9 procedure code(s) on the discharge record, and were classified as surgical, endoscopic, or radiologic. Among patients undergoing procedural intervention, we evaluated the incidence of all relevant post-procedural AEs, including wound-related, infectious, urinary, pulmonary, cardiovascular, gastrointestinal, and intra/peri-operative events (Supplementary Table 3).

The primary exposure of interest was whether patients were admitted to rural or urban hospitals. The classification of rural or urban hospital location used the Core-Based Statistical Area (CBSA) codes, based on the 2000 US Census data from 2005 to 2013 NIS data or the 2010 US Census data for 2014 NIS data. Hospitals with CBSA codes defined as “Division” or “Metro” were classified as urban, and those with codes of “Rural” or “Micropolitan” were classified as rural.

Other covariables included patient age, sex, race (white, black, Hispanic, Asian, or other), primary method of payment (Medicare, Medicaid, private insurance, and other, which included self-pay, no charge, and worker’s compensation), median household income for the ZIP code of residence (based on quartiles), hospital region (Northeast, Midwest, South, and West), weekend admission status, year of admission, and chronic comorbidities. The Elixhauser comorbidity index was used to measure the overall burden of comorbid conditions. Separate sensitivity analysis was additionally conducted utilizing the Charlson enhanced comorbidity index (Supplementary Tables 4 & 5).

### Statistical analysis

All analyses were adjusted for the complex survey design. Revised trend weights accounting for changes in sampling over time were applied to ensure comparable, nationally representative estimates(16). Sampling weights were used to account for the NIS sampling design. Variance estimates were made using the Taylor linearization method to reflect the survey design. Unadjusted comparisons between urban and rural hospitals were made using the survey-adjusted Pearson χ^2^ test, adjusted Wald test, and univariable logistic regression as appropriate. Temporal trends in choledocholithiasis and cholangitis hospitalizations were evaluated by calculating the annual percent change (APC) using a generalized linear model that assumes a Poisson distribution. Average APCs (AAPC) were considered statistically significant when the 95% confidence interval (CI) did not cross 0. Joinpoint regression was then used to assess statistical inflection points in temporal trends.

Survey-adjusted multivariable logistic regression models were used to evaluate the independent association between urban-rural hospital classification and clinical outcomes of interest. Potential confounders selected *a priori* included age, sex, race, primary method of payment, comorbidity burden, weekend admission, year of admission, hospital teaching status, median household income, and hospital region. Survey-adjusted Poisson regression analyses were also used to evaluate mean differences (and 95% Cis) in LOS and hospital charges between urban and rural hospitals.

Survey-adjusted analyses were conducted using Stata (version 17/MP, StataCorp LLC, College Station, TX) and joinpoint analyses were conducted using Joinpoint Regression Program 4.9.0.1 (February 2022, Statistical Methodology and Applications Branch, Surveillance Research Program, National Cancer Institute).(17).

## Results

### Study population

In total, 77,394,755 unweighted discharges were sampled from the NIS between 2005 and 2014 inclusive. After excluding pediatric patients and those with secondary and/or subsequent diagnosis code(s) describing malignancy, 168,838 and 20,524 discharges (unweighted) were identified between 2005 and 2014 by a primary diagnosis code of symptomatic choledocholithiasis and cholangitis, respectively.

Baseline patient demographic and hospital-related characteristics from the NIS 2014 sample are described in Table 1. A total of 16,996 (unweighted) and 1987 (unweighted) patients were identified with a primary discharge diagnosis of choledocholithiasis and cholangitis, respectively. The average age of patients with choledocholithiasis and cholangitis was 57.5 (standard deviation, SD, 20.8) years and 63.0 (SD 17.9) years, respectively. 63.4% of patients with choledocholithiasis were female, while only 48.8% of those with cholangitis were female.

**Table 1:** Survey-weighted baseline patient demographic and hospital-related characteristics from sampled discharges with a primary diagnosis of choledocholithiasis and cholangitis requiring admission, National Inpatient Sample 2014

Characteristic	Primary Discharge Diagnosis	All Hospital Admissions [95% CI]	Admissions to Rural Hospitals [95% CI]	Admissions to Urban Hospitals [95% CI]	p-value
Weighted discharges	Choledocholithiasis	84,980 [82,564–87,396]	6060 [5502–6618]	78,920 [76,569–81,271]	-
	Cholangitis	9935 [9202–10,668]	705 [585–825]	9230 [8507–9953]	-
Mean age (SD)	Choledocholithiasis	57.5 [20.8]	59.1 [21.2]	57.4 [20.8]	0.011
	Cholangitis	63.0 [17.9]	67.1 [17.0]	62.7 [17.9]	0.0033
Female Sex (%)	Choledocholithiasis	63.4 [62.6–64.2]	60.2 [57.4–63.0]	63.7 [62.8–64.5]	0.018
	Cholangitis	48.8 [46.5–51.1]	56.0 [48.0–63.7]	48.3 [45.9–50.7]	0.068
**Race (%)**					
White	Choledocholithiasis	65.7 [64.3–67.1]	83.6 [80.5–86.2]	64.4 [62.9–65.9]	< 0.00001
Black		8.8 [8.2–9.4]	5.4 [4.1–7.2]	9.0 [8.4–9.7]	
Hispanic		17.8 [16.5–19.1]	5.4 [4.0–7.3]	18.7 [17.4–20.1]	
Asian or Pacific Islander		3.5 [3.1–3.9]	1.8 [0.9–3.5]	3.6 [3.2–4.1]	
Other		4.2 [3.8–4.7]	3.7 [2.4–5.8]	4.3 [3.8–4.8]	
White	Cholangitis	72.0 [69.4–74.5]	85.4 [77.8–90.6]	71.1 [68.4–73.7]	0.020
Black		9.4 [8.0–11.0]	4.9 [2.2–10.5]	9.7 [8.3–11.4]	
Hispanic		10.7 [9.1–12.5]	6.5 [3.3–12.5]	11.0 [9.3–12.9]	
Asian or Pacific Islander		5.0 [4.0–6.2]	2.4 [0.8–7.3]	5.2 [4.2–6.5]	
Other		2.9 [2.2–3.8]	0.8 [0.1–5.6]	3.0 [2.3–4.0]	
Other		4.0 [3.5–4.5]	3.8 [2.8–5.3]	4.0 [3.5–4.6]	
**Payment** **method (%)**					
Medicare	Choledocholithiasis	42.9 [42.0–43.9]	50.1 [47.0–53.1]	42.4 [41.4–43.3]	< 0.0001
Medicaid		15.9 [15.0–16.7]	14.3 [12.5–16.3]	16.0 [15.1–16.9]	
Private Insurance		30.6 [29.8–31.5]	25.5 [22.9–28.3]	31.0 [30.1–31.9]	
Other		10.6 [10.0–11.2]	10.1 [8.4–12.1]	10.6 [10.0–11.3]	
Medicare	Cholangitis	54.2 [51.8–56.5]	68.8 [60.6–75.9]	53.1 [50.7–55.5]	< 0.001
Medicaid		9.9 [8.5–11.5]	2.1 [0.7–6.4]	10.5 [8.9–12.3]	
Private Insurance		30.7 [28.5–32.9]	22.7 [16.5–30.3]	31.3 [29.0–33.6]	
Other		5.2 [4.3–6.4]	6.4 [3.3–11.9]	5.2 [4.2–6.3]	
**Median household income of ** **ZIP code of residence (%)**					
<$24,999	Choledocholithiasis	28.0 [26.8–29.3]	44.2 [40.0–48.6]	26.8 [25.5–28.1]	< 0.00001
$25,000–34,999		28.3 [27.3–29.4]	40.6 [36.8–44.5]	27.4 [26.4–28.5]	
$35,000–44,999		23.5 [22.6–24.4]	13.0 [10.8–15.4]	24.3 [23.3–25.3]	
≥$45,000		20.2 [19.0–21.4]	2.2 [1.3–3.6]	21.5 [20.3–22.8]	
<$24,999	Cholangitis	23.6 [21.4–25.9]	42.0 [33.6–50.9]	22.1 [19.9–24.5]	< 0.00001
$25,000–34,999		26.8 [24.6–29.2]	44.2 [35.8–52.9]	25.5 [23.2–27.9]	
$35,000–44,999		23.5 [21.6–25.6]	9.4 [5.6–15.5]	24.6 [22.5–26.8]	
≥$45,000		26.1 [23.5–28.9]	4.3 [2.0–9.4]	27.8 [25.0–30.7]	
**Chronic Conditions (%)**					
None	Choledocholithiasis	51.1 [50.3–52.0]	50.7 [47.9–53.6]	51.2 [50.3–52.0]	0.95
1 chronic comorbidity		23.9 [23.3–24.6]	24.2 [21.8–26.7]	23.9 [23.3–24.6]	
2 chronic comorbidities		11.4 [11.0–11.9]	11.9 [10.2–13.8]	11.4 [10.9–11.9]	
≥ 3 chronic comorbidities		13.5 [12.9–14.0]	13.2 [11.4–15.2]	13.5 [12.9–14.1]	
None	Cholangitis	27.5 [25.5–29.6]	31.2 [24.4–38.9]	27.2 [25.1–29.4]	0.23
1 chronic comorbidity		22.1 [20.4–24.0]	25.5 [19.0–33.4]	21.9 [20.1–23.8]	
2 chronic comorbidities		16.4 [14.8–18.1]	17.0 [11.7–24.1]	16.3 [14.7–18.1]	
≥ 3 chronic comorbidities		34.0 [31.7–36.5]	26.2 [19.3–34.6]	34.6 [32.1–37.2]	
Weekend Admission (%)	Choledocholithiasis	25.0 [24.3–25.7]	22.9 [20.7–25.3]	25.1 [24.5–25.9]	0.076
	Cholangitis	23.8 [21.9–25.8]	21.3 [15.3–28.8]	24.0 [22.1–26.0]	0.47
**Hospital Region (%)**					
Northeast	Choledocholithiasis	18.3 [17.3–19.4]	14.0 [11.5–17.0]	18.7 [17.6–19.8]	< 0.00001
Midwest		20.5 [19.3–21.6]	27.6 [23.7–31.9]	19.9 [18.7–21.1]	
South		36.1 [34.7–37.5]	45.1 [40.5–49.8]	35.4 [34.0–36.8]	
West		25.1 [23.9–26.4]	13.2 [10.7–16.1]	26.0 [24.7–27.4]	
Northeast	Cholangitis	21.9 [19.0–25.2]	12.8 [8.6–18.6]	22.6 [19.5–26.1]	< 0.0001
Midwest		22.8 [19.8–26.1]	36.2 [28.4–44.8]	21.8 [18.6–25.3]	
South		32.9 [29.6–36.4]	39.7 [31.6–48.4]	32.4 [28.9–36.1]	
West		22.3 [19.5–25.5]	11.3 [7.3–17.2]	23.2 [20.1–26.6]	

Approximately 92% of discharges for choledocholithiasis and cholangitis occurred in urban hospitals. In urban hospitals, there was a higher proportion of younger, non-white patients with either Medicaid/private insurance, and with higher relative income quartiles for both patients hospitalized with choledocholithiasis and cholangitis (all p-values < 0.05). No differences were observed in weekend admission status or chronic comorbidity burden between urban and rural hospitals for patients with choledocholithiasis or cholangitis.

### Temporal trends in choledocholithiasis and cholangitis

**Fig. 1:**
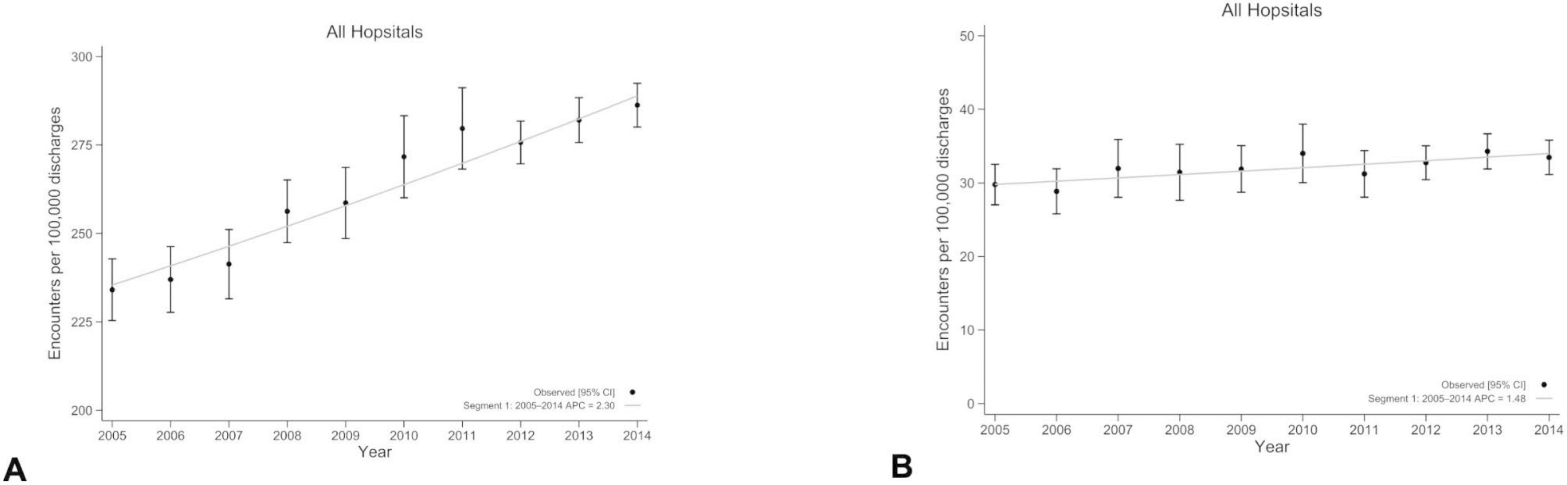
Temporal change in rates of hospital admissions for Choledocholithiasis **(A)** and cholangitis **(B)** per 10,000 hospitalizations with estimated annual percent change and joinpoint regression among all hospital admissions

**Fig. 2:**
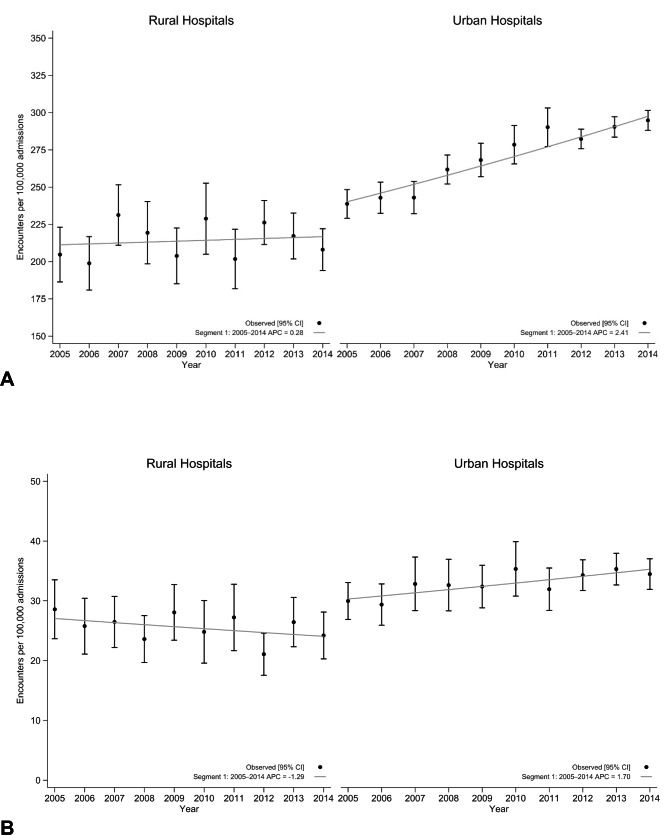
Annual trends in hospital admissions for Choledocholithiasis **(A)** and cholangitis **(B)** with estimated annual percent change and joinpoint regression among admissions to rural (left) and urban (right) census division hospitals

From 2005 to 2014, the estimated national prevalence rates of choledocholithiasis per 100,000 hospital admissions rose steadily from 234.1 (95% CI: 225.4, 242.8) in 2005 to 286.3 (95% CI: 280.1, 292.5) by 2014. For cholangitis, the estimated national prevalence rates per 100,000 hospital admissions rose slightly from 29.8 (95% CI: 27.0, 32.5) in 2005 to 33.5 (95% CI: 31.1, 35.8) by 2014. Temporal trends for choledocholithiasis and cholangitis discharges between 2005 and 2014 are displayed in Figs. 1 and 2. For choledocholithiasis, the overall rate of hospital admissions rose steadily from 2005 to 2014 (AAPC 2.3%, 95% CI 1.9–2.7%). Subdivided by rural and urban regions, the rate of hospital admissions at rural hospitals was stable during the study period (AAPC 0.3%, 95% CI -1.1–1.7%), however, increased in urban hospitals (AAPC 2.4%, 95% CI 1.9–2.9%).

The overall rate of admissions for cholangitis rose between 2005 and 2014 (AAPC 1.5%, 95% CI 0.7–2.2%). The rate of admissions at rural hospitals were stable during this period (AAPC − 1.3%, 95% CI -3.4–0.9%). Conversely, admissions for cholangitis increased in urban hospitals (AAPC 1.7%, 95% CI 0.9–2.6%) during the same period.

### In-hospital mortality

Predictors of in-hospital mortality are summarized in Table 2. There were no significant differences in mortality between rural and urban centers after adjusting for covariables for both choledocholithiasis (aOR 1.16, 95% CI 0.89 to 1.52, p = 0.27) and cholangitis (aOR 1.12, 95% CI 0.72 to 1.72, p = 0.62). Older age was highly associated with mortality in patients with choledocholithiasis, with aOR of 3.56 (95% CI 1.84 to 6.87, p < 0.001) for patients 40–64 years of age and aOR 8.16 (95% CI 4.15 to 16.07, p < 0.001) for patients > 65 years of age compared to those < 40 years of age. Age was not significantly associated with mortality in patients with cholangitis. The presence and number of comorbidities was a strong predictor of mortality for patients with both choledocholithiasis (aOR 15.17, 95% CI 7.98 to 28.82, p < 0.001 for patients with ≥ 3 comorbidities compared to none) and cholangitis (aOR 5.95, 95% CI 2.52 to 14.06, p < 0.001 for patients with ≥ 3 comorbidities compared to none). Lastly, in-hospital mortality had a significant decrease per year over the study period for both patients with choledocholithiasis (aOR 0.90, 95% CI 0.88 to 0.93, p < 0.001) and cholangitis (aOR 0.93, 95% CI 0.89 to 0.97, p < 0.001).

**Table 2:** Univariate and multivariate analysis for predictors of in-hospital mortality in patients admitted for choledocholithiasis and cholangitis

Characteristic		Unadjusted OR [95% CI]	Unadjusted OR p-value	Adjusted OR [95% CI]	Adjusted OR p-value
Female sex	Choledocholithiasis	0.60 [0.53–0.69]	< 0.00001	0.76 [0.66–0.88]	< 0.001
	Cholangitis	1.06 [0.86–1.32]	0.56	0.98 [0.78–1.24]	0.89
Weekend Admission	Choledocholithiasis	0.93 [0.79–1.09]	0.37	0.96 [0.80–1.14]	0.63
	Cholangitis	0.89 [0.69–1.15]	0.39	0.88 [0.66–1.16]	0.36
Year of Admission	Choledocholithiasis	0.93 [0.91–0.95]	< 0.00001	0.90 [0.88–0.93]	< 0.00001
	Cholangitis	0.94 [0.91–0.98]	0.0047	0.93 [0.89–0.97]	< 0.001
**Age**					
< 40 Years	Choledocholithiasis	1	.	1	.
	Cholangitis	1	.	1	.
40–64 years	Choledocholithiasis	6.67 [3.82–11.64]	< 0.00001	3.56 [1.84–6.87]	< 0.001
	Cholangitis	1.90 [1.07–3.36]	0.027	1.10 [0.59–2.04]	0.45
> 65 Years	Choledocholithiasis	31.91 [18.79–54.20]	< 0.00001	8.16 [4.15–16.07]	< 0.00001
	Cholangitis	3.73 [2.16–6.42]	< 0.00001	1.81 [0.93–3.52]	0.082
**Race**					
White	Choledocholithiasis	1	-	1	-
	Cholangitis	1	-	1	-
Black	Choledocholithiasis	0.86 [0.65–1.13]	0.28	1.30 [0.98–1.72]	0.068
	Cholangitis	1.56 [1.05–2.30]	0.026	1.71 [1.13–2.59]	0.011
Hispanic	Choledocholithiasis	0.56 [0.45–0.71]	< 0.00001	1.15 [0.90–1.47]	0.26
	Cholangitis	1.17 [0.79–1.73]	0.43	1.37 [0.92–2.04]	0.12
Asian or Pacific Islander	Choledocholithiasis	0.85 [0.57–1.27]	0.42	0.98 [0.65–1.47]	0.91
	Cholangitis	1.47 [0.93–2.34]	0.100	1.51 [0.95–2.40]	0.079
Other	Choledocholithiasis	0.44 [0.27–0.72]	0.0010	0.67 [0.41–1.09]	0.11
	Cholangitis	1.24 [0.68–2.28]	0.48	1.44 [0.78–2.66]	0.25
**Payment Method**					
Medicare	Choledocholithiasis	1	-	1	-
	Cholangitis	1	-	1	-
Medicaid	Choledocholithiasis	0.19 [0.14–0.26]	< 0.00001	1.05 [0.72–1.53]	0.80
	Cholangitis	0.73 [0.48–1.10]	0.13	1.13 [0.69–1.86]	0.63
Private Insurance	Choledocholithiasis	0.16 [0.12–0.19]	< 0.00001	0.54 [0.39–0.75]	< 0.001
	Cholangitis	0.51 [0.38–0.67]	< 0.00001	0.83 [0.57–1.21]	0.34
Other	Choledocholithiasis	0.14 [0.10–0.20]	< 0.00001	0.77 [0.50–1.18]	0.23
	Cholangitis	0.53 [0.31–0.89]	0.017	0.85 [0.43–1.69]	0.64
**Comorbid Conditions**					
None	Choledocholithiasis	1	-	1	-
	Cholangitis	1	-	1	-
1 Comorbidity	Choledocholithiasis	2.72 [2.19–3.38]	< 0.00001	2.99 [1.52–5.86]	0.0015
	Cholangitis	1.98 [1.34–2.94]	< 0.001	1.67 [0.66–4.24]	0.28
2 Comorbidities	Choledocholithiasis	6.39 [5.12–7.98]	< 0.00001	4.43 [2.32–8.46]	< 0.00001
	Cholangitis	2.25 [1.49–3.41]	< 0.001	2.82 [1.15–6.89]	0.023
≥ 3 Comorbidities	Choledocholithiasis	12.88 [10.67–15.54]	< 0.00001	15.17 [7.98–28.82]	< 0.00001
	Cholangitis	4.75 [3.41–6.62]	< 0.00001	5.95 [2.52–14.06]	< 0.00001
**Urban Vs. Rural Hospital**					
Urban Hospital	Choledocholithiasis	0.99 [0.79–1.24]	0.92	1.16 [0.89–1.52]	0.27
	Cholangitis	1.01 [0.71–1.45]	0.94	1.12 [0.72–1.72]	0.62

### Endoscopic, radiographic, and surgical interventions and adverse events

The use of surgical, radiologic, and endoscopic interventions in patients with choledocholithiasis and cholangitis is summarized in Table 3, stratified by urban and rural divisions. All multivariate models were adjusted for for age category, patient sex, race, insurance status, income quartile, Elixhauser comorbdity burden category/Charlson comorbdity burden category, and weekend hospital admission. The use of any procedural intervention was higher in urban centers for both choledocholithiasis (90.9% vs. 77.5%, aOR = 2.94, 95% CI 2.72 to 3.17; p < 0.001) and cholangitis (55.0% vs. 27.8%, aOR = 2.97, 95% CI: 2.50 to 3.54; p < 0.001) compared to rural hospitals.

**Table 3:** Unadjusted and adjusted risk of procedural interventions, post-operative adverse events, and in-hospital post-intervention mortality between urban and rural hospitals for patients admitted with choledocholithiasis and cholangitis

Outcome		Unadjusted Risk Rural Hospital % [95% CI]	Unadjusted Risk Urban Hospital % [95% CI]	Unadjusted OR [95% CI]	Unadjusted OR p-value	Adjusted OR* [95% CI]	Adjusted OR p-value
**All-cause in-hospital mortality**	Choledocholithiasis	0.5 [0.4–0.6]	0.5 [0.5–0.6]	0.99 [0.79–1.24]	0.92	1.24 [0.94–1.64]	0.13
	Cholangitis	1.7 [1.1–2.2]	1.7 [1.5–1.9]	1.01 [0.71–1.45]	0.94	1.15 [0.74–1.80]	0.54
**Any intervention** ^**Δ**^	Choledocholithiasis	77.5 [76.5–78.5]	90.9 [90.7–91.1]	2.90 [2.72–3.10]	< 0.00001	2.94 [2.72–3.17]	< 0.00001
	Cholangitis	27.8 [25.0–30.6]	55.0 [54.1–55.9]	3.17 [2.75–3.66]	< 0.00001	2.97 [2.50–3.54]	< 0.00001
Any surgical intervention	Choledocholithiasis	59.3 [58.2–60.5]	58.4 [57.9–58.8]	0.96 [0.91–1.01]	0.12	0.96 [0.91–1.02]	0.24
	Cholangitis	6.7 [5.5–7.8]	7.3 [6.9–7.7]	1.10 [0.91–1.35]	0.32	1.12 [0.89–1.41]	0.34
Any radiologic intervention	Choledocholithiasis	0.7 [0.5–0.8]	1.9 [1.8–2.0]	2.90 [2.35–3.58]	< 0.00001	2.79 [2.20–3.54]	< 0.00001
	Cholangitis	1.0 [0.5–1.4]	6.5 [6.1–7.0]	7.26 [4.53–11.63]	< 0.00001	5.78 [3.43–9.74]	< 0.00001
Any endoscopic intervention	Choledocholithiasis	44.8 [43.0–46.7]	75.3 [74.9–75.6]	3.75 [3.47–4.05]	< 0.00001	3.61 [3.33–3.94]	< 0.00001
	Cholangitis	23.8 [21.1–26.5]	47.8 [46.8–48.8]	2.93 [2.51–3.43]	< 0.00001	2.79 [2.32–3.36]	< 0.00001
**Any post-intervention adverse event**	Choledocholithiasis	14.4 [13.8–15.1]	15.3 [15.1–15.5]	1.07 [1.01–1.13]	0.019	1.10 [1.03–1.18]	0.0047
	Cholangitis	33.8 [29.5–38.1]	35.4 [34.4–36.4]	1.07 [0.88–1.30]	0.49	1.01 [0.80–1.28]	0.94
Any post-operative complication adverse event	Choledocholithiasis	14.9 [14.1–15.6]	15.9 [15.6–16.1]	1.08 [1.01–1.15]	0.021	1.14 [1.05–1.23]	< 0.001
	Cholangitis	40.3 [31.1–49.5]	45.2 [42.6–47.9]	1.22 [0.82–1.82]	0.32	1.10 [0.68–1.77]	0.70
Any post-radiologic complication adverse event	Choledocholithiasis	28.1 [19.3–36.9]	33.1 [31.4–34.9]	1.27 [0.82–1.98]	0.29	1.37 [0.84–2.23]	0.21
	Cholangitis	59.7 [39.7–79.7]	47.7 [44.7–50.7]	0.62 [0.27–1.42]	0.26	0.76 [0.28–2.09]	0.60
Any post-endoscopic complication adverse event	Choledocholithiasis	14.1 [13.2–15.0]	15.1 [14.9–15.4]	1.09 [1.01–1.17]	0.028	1.15 [1.05–1.26]	0.0022
	Cholangitis	32.5 [27.9–37.1]	33.8 [32.8–34.9]	1.06 [0.86–1.32]	0.57	1.03 [0.80–1.34]	0.79
**Any post-intervention mortality**	Choledocholithiasis	0.5 [0.4–0.6]	0.5 [0.5–0.5]	1.00 [0.77–1.29]	0.97	1.10 [0.81–1.50]	0.55
	Cholangitis	1.5 [0.5–2.5]	1.5 [1.3–1.8]	1.01 [0.51–1.99]	0.98	2.02 [0.71–5.70]	0.19
Post-operative mortality	Choledocholithiasis	0.4 [0.3–0.6]	0.5 [0.4–0.5]	1.05 [0.77–1.45]	0.75	1.19 [0.81–1.74]	0.38
	Cholangitis	2.9 [0.1–5.7]	1.9 [1.2–2.6]	0.66 [0.22–1.94]	0.45	4.33 [0.57–32.90]	0.16
Post-radiologic mortality	Choledocholithiasis	4.3 [0.6–8.0]	2.6 [2.0–3.2]	0.60 [0.24–1.52]	0.28	0.65 [0.22–1.86]	0.42
	Cholangitis	8.8 [0.0–20.2]*	3.4 [2.4–4.5]	0.37 [0.09–1.62]	0.19	0.92 [0.12–7.20]	0.94
Post-endoscopic mortality	Choledocholithiasis	0.5 [0.3–0.6]	0.4 [0.4–0.5]	0.87 [0.62–1.22]	0.41	0.90 [0.62–1.33]	0.61
	Cholangitis	1.4 [0.4–2.4]	1.4 [1.1–1.6]	0.99 [0.46–2.14]	0.98	2.05 [0.61–6.85]	0.24

* Models were adjusted for age category, patient sex, race, insurance status, income quartile, Elixhauser burden category, and weekend hospital admission

^Δ^ Intervention refers to the use of any procedural intervention, including surgery/endoscopy/interventional radiology

Endoscopic interventions were the most common procedural intervention performed overall for both choledocholithiasis and cholangitis in urban hospitals. However, surgical interventions (59.3%, 95% CI 58.2–60.5%) were more commonly performed compared to endoscopy (44.8%, 95% CI 43.0–46.7%) for choledocholithiasis in rural centers. After adjusting for covariables, practitioners at urban hospitals were more likely to perform radiological interventions (aOR 2.79, 95% CI 2.20 to 3.54, p < 0.001) and endoscopic interventions (aOR 3.61, 95% CI 3.33 to 3.94, p < 0.001) for patients with choledocholithiasis. Similarly, amongst those with cholangitis, practitioners at urban centers were more likely to perform radiological (aOR 5.78, 95% CI 3.43 to 9.74, p < 0.001) and endoscopic interventions (aOR 2.79, 95% CI 2.32 to 3.36, p < 0.001). The likelihood of performing surgical interventions did not differ between rural and urban centers for both choledocholithiasis and cholangitis.

Overall, post-intervention AEs were moderately common amongst patients with choledocholithiasis (complication rate 1523 [95% CI 1502 to 1545] per 10,000 discharges associated with an intervention) and cholangitis (complication rate 3534 [95% CI 3437 to 3632] per 10,000) amongst those undergoing procedures. Post-intervention gastrointestinal AEs (497 [95% CI 484–509] per 10,000) were the most common amongst patients with choledocholithiasis, followed by post-intervention infectious complications (431 [95% CI 420 to 443] per 10,000). However, post-intervention infectious AEs (2417 [95% CI 2330 to 2507] per 10,000) the most common among patients with cholangitis, followed by post-intervention gastrointestinal AEs (417 [95% CI 380 to 458] per 10,000). Rates of post-intervention AEs stratified by subtype are summarized in the Supplementary Materials.

Amongst patients undergoing procedural interventions, rates of any post-intervention AEs were slightly more frequent at urban hospitals amongst patients with choledocholithiasis (aOR 1.13, 95% CI 1.03 to 1.18, p = 0.0047), however, were not significantly different for patients with cholangitis (aOR 1.01, 95% CI 0.80 to 1.28, p = 0.94). Subdivided by intervention type, surgical post-intervention AEs (aOR 1.14, 95% CI 1.05 to 1.23, p < 0.001) and post-endoscopic AEs (aOR 1.15, 95% CI 1.05 to 1.26, p = 0.0022) were slightly increased in patients with choledocholithiasis admitted to urban hospitals. There were otherwise no statistically significant differences in AE rates between urban and rural hospitals based on intervention type. There were no differences in post-intervention mortality between rural and urban hospitals for both choledocholithiasis (aOR 1.10, 95% CI 0.81 to 1.50, p = 0.55) and cholangitis (aOR 2.02, 95% CI 0.71 to 5.70, p = 0.19).

## Discussion

In our analysis of over 180,000 unweighted discharges for patients with choledocholithiasis and cholangitis in the NIS between 2005 and 2014, we found a rising incidence for both choledocholithiasis and cholangitis, particularly in urban regions. We also demonstrated that age and presence of comorbidities were strong predictors of in-hospital mortality, however, residing in a rural location was not significantly associated with mortality. There were, however, significant differences between urban and rural regions in terms of use of procedural interventions and post-intervention AEs. Notably, urban centers had an overall higher rate of use of procedural interventions and an increased risk of post-intervention AEs for patients with choledocholithiasis.

The steady increase in the prevalence of choledocholithiasis and cholangitis during our study period is expected as other studies have shown an overall rising prevalence of cholelithiasis in the United States for which we would expect choledocholithiasis and cholangitis to parallel [18, 19]. This rise may be related to increasing rates of established risk factors in developed western nations, including obesity, metabolic syndrome, older age, and rapid weight loss [20]. Although studies have shown an overall increasing prevalence trend of cholelithiasis, it should be noted that this is driven primarily by ambulatory and emergency department visits [18]. The rates of cholelithiasis and cholecystitis requiring hospitalization in contrast are decreasing in the U.S, with multiple studies demonstrating between a 5 and 13% decrease in hospitalizations between 2005 and 2014(18, 21). This is likely related to the increasing use and availability of ambulatory laparoscopic cholecystectomy in the U.S [19]. Interestingly, our trends for hospital discharges for choledocholithiasis and cholangitis do not mimic that for cholelithiasis and cholecystitis as would have been expected, and instead showed a steady increase during this period. In addition, our estimates of total discharges for choledocholithiasis and cholangitis are likely an underestimate as we maximized specificity in our selected patient population by only including those with a primary ICD-9 code of choledocholithiasis and/or cholangitis, thus potentially excluding individuals with secondary diagnoses of the same.

There are likely several reasons for this trend. First, the clinical presentation of gallstone disease can vary significantly. Patients with biliary colic generally appear well and do not have biochemical abnormalities or systemic findings such as fever or jaundice, unlike those with choledocholithiasis or cholangitis [22]. Additionally, patients with choledocholithiasis may have more severe presentations due to associated conditions such as gallstone pancreatitis and cholangitis [23]. As such, it may be hypothesized that differences in severity of presentation may result in patients with symptomatic cholelithiasis to be more likely managed in the outpatient setting, while patients presenting with symptomatic choledocholithiasis are more likely to be hospitalized. Secondly, although cholecystectomy in general eliminates the future recurrence of symptomatic cholelithiasis or cholecystitis, this is not always the case for choledocholithiasis or cholangitis [24]. The majority of choledocholithiasis is related to secondary choledocholithiasis, due to gallstones migrating from the gallbladder into the common bile duct for which total cholecystectomy should prevent [25, 26]. However, there have been higher rates of subtotal cholecystectomies in the past decade [27, 28]. Patients might therefore experience recurrent gallbladder stones forming in a gallbladder or long cystic duct remnant that may result in choledocholithiasis and/or cholangitis in the future [27]. Recurrent symptomatic gallstone disease after incomplete cholecystectomy can be common and may require completion cholecystectomy and/or cystic duct revision to prevent future recurrence [27].

In addition, although up to 12% of patients may have associated common bile duct stones (CBDS) at the time of laparoscopic cholecystectomy, the use of routine intraoperative cholangiograms (IOC) has been controversial, and in past decades has significantly decreased in use [29, 30]. A recent meta-analysis reported that routine use of IOC can detect over 3 times the number of CBDS compared to selective IOC [29]. Notably, several studies have shown increased rates of future biliary complications if asymptomatic or incidental CBDS were left in place [31, 32]. Lastly, a significant proportion of patients may have primary choledocholithiasis, related to stones forming directly within the intra or extrahepatic bile ducts [26]. Primary stones have been identified as a major cause of choledocholithiasis and/or cholangitis even after cholecystectomy and ERCP [26, 33]. Identified risk factors include anatomic abnormalities (strictures, peri-ampullar diverticulum), advanced age, and bacterial infections. A 2% rate of recurrent choledocholithiasis with the use of surgical CBDE has been demonstrated compared to 8.9% for ERCP, presumably due to preservation of the sphincter of Oddi via CBDE thus preventing reflux of intestinal microbial contents into the biliary system [34]. With ERCP having supplanted surgical CBDE as the preferred procedure for choledocholithiasis in most institutions over the past decades, this may have increased the incidence of primary recurrent choledocholithiasis.

Comparing urban and rural centers, we observed major differences in patient demographics and use of procedural interventions; however, in-hospital and post-intervention mortality were similar between groups. There was a significantly higher rate of all procedural interventions in urban hospitals for both choledocholithiasis and cholangitis, likely reflecting an increased availability of resources compared to rural hospitals. This is in line with the 2010 report by the National Center for Health Statistics where 64% of rural inpatients did not receive procedural interventions, versus 38% of urban inpatients [35]. Interestingly, endoscopic interventions were the most common procedural intervention for patients with choledocholithiasis (75.3% endoscopic vs. 58.4% surgical vs. 1.9% radiologic) at urban hospitals, however, surgical interventions were most common (59.3% surgical vs. 44.8% endoscopic vs. 0.7% radiologic) at rural hospitals. This is likely related to the limited availability of ERCP in rural areas, which ultimately may require patients to be transferred to urban hospitals to receive ERCP followed by subsequent cholecystectomy at their rural hospital [14, 36]. Absolute rates of surgical intervention were similar between urban and rural sites, supporting that general access to cholecystectomy in rural centers is comparable to urban centers. Notably, our analysis did not account for transfers between urban and rural centers, thus the differences in intervention rates may in part reflect inter-facility transfers from rural to urban centers to have procedures performed due to lack of local resources or expertise. Lastly. in line with recommendations that radiologic percutaneous biliary drainage is not preferred compared to endoscopic/surgical management, we found that radiologic interventions were the least common intervention in both urban and rural areas [2, 7].

Interestingly, Poulose et al. had also shown that rural centers had a higher proportion of patients undergoing surgical CBDE compared to urban centers, although the absolute number was small [14]. Wandling et al. have found however with the NIS, that ERCP with subsequent laparoscopic cholecystectomy has overwhelmingly become the preferred management option over the recent decade, with sharp declines in the use of CBDE [37]. However, as the wide-spread implementation of ERCP is challenging due to the need for high-volumes to maintain ERCP skills and quality, the use of L-CBDE may be an alternative option in rural areas [14]. L-CBDE has shown comparable efficacy and advantages compared to ERCP, such as decreased length of stay and only requiring a single-stage procedure [38, 39]. Despite this, modern surgical training programs have seen a significant decline in exposure to CBDE in recent decades, and many surgeons may not be comfortable in performing this directly out of surgical training [40]. However, Campagna et al. had recently demonstrated that experienced rural general surgeons could gain and maintain procedural confidence in L-CBDE after a dedicated short-term training course [41]. But even with adequate experience, availability of required equipment for L-CBDE and knowledgeable OR staff may still limit practice in rural areas [41]. Thus, it is key that the preferred management pathway for choledocholithiasis be tailored to what local resources and expertise allows in rural areas.

We also found a higher rate of post-intervention AEs in patients at urban hospitals that was driven by post-surgical and post-endoscopic AEs. This is most likely related to increased patient complexity and acuity at urban centers and reflects inter-facility transfers for more ill patients from rural to urban referral centers which we were unable to separate in our analysis. Prior data has suggested that although the overall rate of adverse events such as post-ERCP pancreatitis (PEP) appears to be lowest in rural hospitals, urban hospitals ultimately had lower odds of PEP after adjusting for the level of ERCP intervention [42, 43]. Additionally, Carbonell et al. had found higher risks of AEs for patients undergoing inpatient cholecystectomy at urban hospitals, in line with our results [44]. Overall rates of post-intervention AEs was noted to be moderately common across urban-rural hospitals for both choledocholithiasis and cholangitis. Recent epidemiological data has observed this trend, with increasing rates of PEP and post-ERCP bleeding over the past decade [45, 46]. This may reflect more aggressive and complex procedures as well as changing patient demographics of established risk factors for AEs such as PEP. Despite this, we have found a decreasing mortality trend for both choledocholithiasis and cholangitis year over year, suggesting that there have been overall improvements in procedural techniques and pre/post procedural care.

Our study has several strengths. To our knowledge, it represents the most comprehensive analysis of patients with choledocholithiasis and cholangitis in the US, comprising over 180,000 unweighted discharges over ten years within a geographically diverse, all-payer, nationally representative dataset. Thus, our results are generalizable across all care regions within the US. However, there are limitations of our study that require acknowledgement. Firstly, as with any administrative database study, ICD-9-CM coding errors are possible, and no studies have formally evaluated the validity of these codes in choledocholithiasis and cholangitis. As our study population was limited to those with primary diagnostic codes for choledocholithiasis and cholangitis, it is possible some patients with these listed as secondary codes were not captured. However, we provided a comprehensive inclusion of ICD-9 variations for appropriate primary diagnostic codes (Supplementary tables) and we felt utilizing primary codes would allow for less coding errors and confounders, which is in line with practices from similar studies on this topic(37). To minimize risks of coding errors, we limited our analysis to 2005–2014 to avoid potential errors with overlapping ICD-10-CM codes, given that the NIS transitioned to the ICD-10-CM in 2015. When comparing rural and urban hospitals, we acknowledge that significant potential variations exist in defining and distinguishing between these categories. Our results were not separately adjusted based on other covariables such as hospital size and teaching status, although rurality has clear associations with both these factors. Finally, although we categorized interventions as surgical, endoscopic, or radiologic, we did not evaluate more granular data on the specific type of procedures being performed which may have been of interest to analyze trends of CBDE and ERCP across urban-rural divisions.

## Conclusion

In conclusion, the rate of hospitalizations for choledocholithiasis and cholangitis have increased between 2005 and 2014. This finding may reflect both the overall increasing prevalence of gallstone disease in the U.S, and the increased requirement of inpatient management for those with choledocholithiasis and cholangitis, among other factors. Patients treated at urban hospitals have higher rates of procedural interventions and post-intervention AEs, likely reflecting increased case complexity in urban centers. Overall, there have been significant improvements in mortality associated with choledocholithiasis and cholangitis over the study period, suggesting potentially improved clinical care pathways. Future studies are needed to evaluate disparities in access to procedural care at rural centers and to assess possible measures to address care gaps such as the increased training and use of L-CBDE.

## Electronic supplementary material

Below is the link to the electronic supplementary material.

Supplementary Material 1: Supplementary ICD-9 Coding, Tables, and Prevalance Trends

## Data Availability

The datasets generated and/or analysed during the current study are available publicly in the HCUP-US NIS repository. The HCUP NIS database years from 2005 to 2014 inclusive were utilized in the current study, which are publicly available for purchase request via: https://hcup-us.ahrq.gov/tech_assist/centdist.jsp.

## Abbreviations

AE: Adverse event
AOR: Adjusted odds ratio
APC: Annual percent change
CBSA: Core-Based Statistical Area
CI: Confidence interval
ERCP: Endoscopic retrograde cholangiopancreatography
CBDE: Common bile duct exploration
IOC: Intraoperative cholangiogram
HCUP: Healthcare Cost and Utilization Project
ICD-9-CM: International Classification of Diseases, Ninth Revision, Clinical Modification
LOS: Length of stay
NIS: National Inpatient Sample
OR: Odds ratio
