# Supplementary material for: Epidemiology and outcomes of choledocholithiasis and cholangitis in the United States: trends and urban-rural variations
Source: BMC Gastroenterol. 2023 Jul 27;23:254. doi: 10.1186/s12876-023-02868-3 (PMC10373232; doi:10.1186/s12876-023-02868-3)

**Supplementary Table 1:** **Definitions of choledocholithiasis and cholangitis based on ICD-9-CM diagnosis codes.**

| **Clinical presentation** | **ICD-9-CM code** | **ICD-9 Description** |
| --- | --- | --- |
| Choledocholithiasis | 574.30 | Calculus of bile duct with acute cholecystitis, without mention of obstruction |
|  | 574.31 | Calculus of bile duct with acute cholecystitis, with obstruction |
|  | 574.40 | Calculus of bile duct with other cholecystitis, without mention of obstruction |
|  | 574.41 | Calculus of bile duct with other cholecystitis, with obstruction |
|  | 574.50 | Calculus of bile duct without mention of cholecystitis, without mention of obstruction |
|  | 574.51 | Calculus of bile duct without mention of cholecystitis, with obstruction |
|  | 574.60 | Calculus of gallbladder and bile duct with acute cholecystitis, without mention of obstruction |
|  | 574.61 | Calculus of gallbladder and bile duct with acute cholecystitis, with obstruction |
|  | 574.70 | Calculus of gallbladder and bile duct with other cholecystitis, without mention of obstruction |
|  | 574.71 | Calculus of gallbladder and bile duct with other cholecystitis, with obstruction |
|  | 574.80 | Calculus of gallbladder and bile duct with acute and chronic cholecystitis, without mention of obstruction |
|  | 574.81 | Calculus of gallbladder and bile duct with acute and chronic cholecystitis, with obstruction |
|  | 574.90 | Calculus of gallbladder and bile duct without cholecystitis, without mention of obstruction |
|  | 574.91 | Calculus of gallbladder and bile duct without cholecystitis, with obstruction |
|  | 576.2 | Obstruction of bile duct |
| **Choledocholithiasis only** |  | Primary diagnosis of choledocholithiasis, excludes secondary diagnosis codes for: cholecystitis, acute pancreatitis, and cholangitis, but does not exclude cholelithiasis. |
| **Cholangitis** | 576.1 | Cholangitis |
| **Cholangitis presentation** |  | Primary diagnosis of choledocholithiasis and secondary diagnosis of cholangitis;  or primary diagnosis of cholangitis with anything as secondary |

**Supplementary Table 2: ICD-9-CM codes for diagnosis of malignancy used to exclude patients from analysis.**

| **Code** | **Description** |
| --- | --- |
| 155.0 | Malignant neoplasm of liver, primary |
| 155.1 | Malignant neoplasm of intrahepatic bile ducts |
| 155.2 | Malignant neoplasm of liver, not specified as primary or secondary |
| 156.0 | Malignant neoplasm of gallbladder |
| 156.1 | Malignant neoplasm of extrahepatic bile ducts |
| 156.2 | Malignant neoplasm of ampulla of Vater |
| 156.8 | Malignant neoplasm of other specified sites of gallbladder and extrahepatic bile ducts |
| 156.9 | Malignant neoplasm of biliary tract, part unspecified site |
| 157.0 | Malignant neoplasm of head of pancreas |
| 157.1 | Malignant neoplasm of body of pancreas |
| 157.3 | Malignant neoplasm of pancreatic duct |
| 157.8 | Malignant neoplasm of other specified sites of pancreas |
| 157.9 | Malignant neoplasm of pancreas, part unspecified |
| 158.0 | Malignant neoplasm of retroperitoneum |
| 158.9 | Malignant neoplasm of peritoneum, unspecified |

**Supplementary Table 3. Survey-weighted proportions per 10,000 of adverse events, following procedures performed in patients with a primary diagnosis of choledocholithiasis and cholangitis, overall and by type of complication and type of intervention, NIS 2005–2014.**

| **Type of Adverse Event** | | **Any Intervention [95% CI]** | **Any Surgery**  **[95% CI]** | **Any Radiology**  **[95% CI]** | **Any Endoscopy**  **[95% CI]** |
| --- | --- | --- | --- | --- | --- |
| Any Adverse Event | Choledocholithiasis | 1523.2 [1501.6 – 1545.0] | 1576.1 [1550.5 – 1602.1] | 3296.5 [3125.8 – 3471.7] | 1506.9 [1483.5 – 1530.5] |
|  | Cholangitis | 3534.0 [3437.1 – 3632.2] | 4483.1 [4227.6 – 4741.4] | 4800.6 [4508.2 – 5094.4] | 3381.0 [3278.5 – 3485.0] |
| Post-Intervention wound complication | Choledocholithiasis | 42.5 [39.4 – 45.9] | 50.2 [45.9 – 54.8] | 114.6 [83.0 – 158.0] | 38.0 [34.7 – 41.6] |
|  | Cholangitis | 44.4 [33.4 – 59.0] | 173.5 [117.9 – 254.6] | 97.1 [55.3 – 169.9] | 33.6 [23.5 – 47.9] |
| Post-Intervention infectious complication | Choledocholithiasis | 431.4 [420.2 – 442.9] | 368.7 [356.5 – 381.3] | 1275.2 [1161.1 – 1398.8] | 443.2 [430.7 – 456.1] |
|  | Cholangitis | 2417.3 [2329.8 – 2507.0] | 2753.9 [2527.5 – 2992.5] | 2993.0 [2721.6 – 3279.3] | 2380.5 [2289.2 – 2474.2] |
| Post-Intervention urinary complication | Choledocholithiasis | 41.4 [38.2 – 44.9] | 59.3 [54.5 – 64.5] | 70.4 [46.2 – 107.1] | 37.1 [33.7 – 40.9] |
|  | Cholangitis | 21.5 [14.3 – 32.3] | 93.9 [55.7 – 157.9] | 31.8 [12.0 – 84.2] | 19.5 [12.3 – 30.9] |
| Post-Intervention pulmonary complication | Choledocholithiasis | 264.6 [256.2 – 273.4] | 323.4 [312.1 – 335.1] | 633.2 [550.5 – 727.5] | 240.2 [231.3 – 249.4] |
|  | Cholangitis | 371.1 [335.9 – 409.7] | 885.6 [747.4 – 1046.5] | 626.1 [505.5 – 773.0] | 326.2 [291.2 – 365.2] |
| Post-Intervention gastrointestinal complication | Choledocholithiasis | 496.5 [484.0 – 509.3] | 568.4 [552.4 – 584.8] | 842.1 [748.5 – 946.2] | 498.9 [485.1 – 513.1] |
|  | Cholangitis | 417.2 [379.8 – 458.2] | 707.4 [586.1 – 851.4] | 703.8 [574.5 – 859.5] | 377.3 [340.2 – 418.3] |
| Post-Intervention cardiac complication | Choledocholithiasis | 134.2 [128.2 – 140.6] | 132.7 [125.6 – 140.3] | 353.5 [292.2 – 427.1] | 127.2 [120.7 – 134.0] |
|  | Cholangitis | 269.2 [240.4 – 301.3] | 369.4 [284.8 – 478.0] | 395.9 [304.2 – 513.7] | 249.9 [219.6 – 284.1] |
| Other intra- and peri-intervention complications | Choledocholithiasis | 218.1 [210.1 – 226.3] | 252.6 [242.4 – 263.3] | 308.9 [250.9 – 379.7] | 205.4 [197.0 – 214.2] |
|  | Cholangitis | 165.8 [142.8 – 192.5] | 441.8 [347.6 – 560.0] | 177.4 [117.4 – 267.2] | 159.5 [135.1 – 188.2] |

**Supplementary Table 4. Univariate and multivariate analysis for predictors of in-hospital mortality in patients admitted for choledocholithiasis and cholangitis.**

| **Characteristic** | | **Unadjusted OR [95% CI]** | **Unadjusted OR**  **p-value** | **Adjusted OR [95% CI]** | **Adjusted OR**  **p-value** |
| --- | --- | --- | --- | --- | --- |
| Female sex | Choledocholithiasis | 0.60 [0.53 – 0.69] | <0.00001 | 0.88 [0.76 – 1.02] | 0.078 |
|  | Cholangitis | 1.06 [0.86 – 1.32] | 0.56 | 1.11 [0.88 – 1.40] | 0.39 |
| Weekend Admission | Choledocholithiasis | 0.93 [0.79 – 1.09] | 0.37 | 0.98 [0.82 – 1.16] | 0.79 |
|  | Cholangitis | 0.89 [0.69 – 1.15] | 0.39 | 0.89 [0.67 – 1.18] | 0.42 |
| Year of Admission | Choledocholithiasis | 0.93 [0.91 – 0.95] | <0.00001 | 0.91 [0.88 – 0.93] | <0.00001 |
|  | Cholangitis | 0.94 [0.91 – 0.98] | 0.0047 | 0.93 [0.89 – 0.97] | <0.001 |
| **Age** | | | | | |
| <40 Years | Choledocholithiasis | 1 | . | 1 | . |
|  | Cholangitis | 1 | . | 1 | . |
| 40-64 years | Choledocholithiasis | 6.67 [3.82 – 11.64] | <0.00001 | 5.15 [2.70 – 9.81] | <0.00001 |
|  | Cholangitis | 1.90 [1.07 – 3.36] | 0.027 | 1.26 [0.69 – 2.29] | 0.45 |
| >65 Years | Choledocholithiasis | 31.91 [18.79 – 54.20] | <0.00001 | 12.43 [6.37 – 24.24] | <0.00001 |
|  | Cholangitis | 3.73 [2.16 – 6.42] | <0.00001 | 2.26 [1.19 – 4.30] | 0.013 |
| **Race** | | | | | |
| White | Choledocholithiasis | 1 | - | 1 | - |
|  | Cholangitis | 1 | - | 1 | - |
| Black | Choledocholithiasis | 0.86 [0.65 – 1.13] | 0.28 | 1.23 [0.93 – 1.62] | 0.15 |
|  | Cholangitis | 1.56 [1.05 – 2.30] | 0.026 | 1.60 [1.06 – 2.42] | 0.026 |
| Hispanic | Choledocholithiasis | 0.56 [0.45 – 0.71] | <0.00001 | 1.08 [0.85 – 1.38] | 0.52 |
|  | Cholangitis | 1.17 [0.79 – 1.73] | 0.43 | 1.33 [0.89 – 1.98] | 0.16 |
| Asian or Pacific Islander | Choledocholithiasis | 0.85 [0.57 – 1.27] | 0.42 | 0.90 [0.60 – 1.36] | 0.62 |
|  | Cholangitis | 1.47 [0.93 – 2.34] | 0.100 | 1.42 [0.89 – 2.26] | 0.14 |
| Other | Choledocholithiasis | 0.44 [0.27 – 0.72] | 0.0010 | 0.64 [0.39 – 1.05] | 0.078 |
|  | Cholangitis | 1.24 [0.68 – 2.28] | 0.48 | 1.38 [0.75 – 2.55] | 0.30 |
| **Payment Method** | | | | | |
| Medicare | Choledocholithiasis | 1 | - | 1 | - |
|  | Cholangitis | 1 | - | 1 | - |
| Medicaid | Choledocholithiasis | 0.19 [0.14 – 0.26] | <0.00001 | 1.04 [0.71 – 1.53] | 0.82 |
|  | Cholangitis | 0.73 [0.48 – 1.10] | 0.13 | 1.11 [0.68 – 1.83] | 0.67 |
| Private Insurance | Choledocholithiasis | 0.16 [0.12 – 0.19] | <0.00001 | 0.53 [0.38 – 0.73] | <0.001 |
|  | Cholangitis | 0.51 [0.38 – 0.67] | <0.00001 | 0.77 [0.53 – 1.11] | 0.16 |
| Other | Choledocholithiasis | 0.14 [0.10 – 0.20] | <0.00001 | 0.76 [0.49 – 1.18] | 0.22 |
|  | Cholangitis | 0.53 [0.31 – 0.89] | 0.017 | 0.85 [0.43 – 1.68] | 0.64 |
| **Comorbid Conditions** | | | | | |
| None | Choledocholithiasis | 1 | - | 1 | - |
|  | Cholangitis | 1 | - | 1 | - |
| 1 Comorbidity | Choledocholithiasis | 2.72 [2.19 – 3.38] | <0.00001 | 1.69 [1.32 – 2.16] | <0.0001 |
|  | Cholangitis | 1.98 [1.34 – 2.94] | <0.001 | 2.01 [1.29 – 3.14] | 0.0021 |
| 2 Comorbidities | Choledocholithiasis | 6.39 [5.12 – 7.98] | <0.00001 | 3.17 [2.45 – 4.11] | <0.00001 |
|  | Cholangitis | 2.25 [1.49 – 3.41] | <0.001 | 2.20 [1.37 – 3.52] | 0.0011 |
| ≥3 Comorbidities | Choledocholithiasis | 12.88 [10.67 – 15.54] | <0.00001 | 6.54 [5.23 – 8.19] | <0.00001 |
|  | Cholangitis | 4.75 [3.41 – 6.62] | <0.00001 | 5.30 [3.60 – 7.79] | <0.00001 |
| **Urban Vs. Rural Hospital** | | | | | |
| Urban Hospital | Choledocholithiasis | 0.99 [0.79 – 1.24] | 0.92 | 1.20 [0.92 – 1.57] | 0.19 |
|  | Cholangitis | 1.01 [0.71 – 1.45] | 0.94 | 1.05 [0.68 – 1.62] | 0.83 |

**Supplementary 5.** Unadjusted and adjusted risk of procedural interventions, post-operative adverse events, and in-hospital post-intervention mortality between urban and rural hospitals for patients admitted with choledocholithiasis and cholangitis.

| **Outcome** | | **Unadjusted Risk Rural Hospital**  **% [95% CI]** | **Unadjusted Risk Urban Hospital**  **% [95% CI]** | **Unadjusted OR**  **[95% CI]** | **Unadjusted OR**  **p-value** | **Adjusted OR***  **[95% CI]** | **Adjusted OR**  **p-value** |
| --- | --- | --- | --- | --- | --- | --- | --- |
| **All-cause in-hospital mortality** | Choledocholithiasis | 0.5 [0.4 – 0.6] | 0.5 [0.5 – 0.6] | 0.99 [0.79 – 1.24] | 0.92 | 1.28 [0.97 – 1.69] | 0.080 |
|  | Cholangitis | 1.7 [1.1 – 2.2] | 1.7 [1.5 – 1.9] | 1.01 [0.71 – 1.45] | 0.94 | 1.10 [0.70 – 1.72] | 0.68 |
| **Any intervention^Δ^** | Choledocholithiasis | 77.5 [76.5 – 78.5] | 90.9 [90.7 – 91.1] | 2.90 [2.72 – 3.10] | <0.00001 | 2.95 [2.74 – 3.18] | <0.00001 |
|  | Cholangitis | 27.8 [25.0 – 30.6] | 55.0 [54.1 – 55.9] | 3.17 [2.75 – 3.66] | <0.00001 | 3.07 [2.58 – 3.65] | <0.00001 |
| Any surgical intervention | Choledocholithiasis | 59.3 [58.2 – 60.5] | 58.4 [57.9 – 58.8] | 0.96 [0.91 – 1.01] | 0.12 | 0.96 [0.91 – 1.02] | 0.22 |
|  | Cholangitis | 6.7 [5.5 – 7.8] | 7.3 [6.9 – 7.7] | 1.10 [0.91 – 1.35] | 0.32 | 1.16 [0.92 – 1.46] | 0.20 |
| Any radiologic intervention | Choledocholithiasis | 0.7 [0.5 – 0.8] | 1.9 [1.8 – 2.0] | 2.90 [2.35 – 3.58] | <0.00001 | 2.82 [2.23 – 3.58] | <0.00001 |
|  | Cholangitis | 1.0 [0.5 – 1.4] | 6.5 [6.1 – 7.0] | 7.26 [4.53 – 11.63] | <0.00001 | 5.60 [3.32 – 9.44] | <0.00001 |
| Any endoscopic intervention | Choledocholithiasis | 44.8 [43.0 – 46.7] | 75.3 [74.9 – 75.6] | 3.75 [3.47 – 4.05] | <0.00001 | 3.62 [3.32 – 3.95] | <0.00001 |
|  | Cholangitis | 23.8 [21.1 – 26.5] | 47.8 [46.8 – 48.8] | 2.93 [2.51 – 3.43] | <0.00001 | 2.89 [2.40 – 3.48] | <0.00001 |
| **Any post-intervention adverse event** | Choledocholithiasis | 14.4 [13.8 – 15.1] | 15.3 [15.1 – 15.5] | 1.07 [1.01 – 1.13] | 0.019 | 1.13 [1.06 – 1.21] | <0.001 |
|  | Cholangitis | 33.8 [29.5 – 38.1] | 35.4 [34.4 – 36.4] | 1.07 [0.88 – 1.30] | 0.49 | 0.99 [0.79 – 1.25] | 0.96 |
| Any post-operative adverse event | Choledocholithiasis | 14.9 [14.1 – 15.6] | 15.9 [15.6 – 16.1] | 1.08 [1.01 – 1.15] | 0.021 | 1.17 [1.09 – 1.26] | <0.0001 |
|  | Cholangitis | 40.3 [31.1 – 49.5] | 45.2 [42.6 – 47.9] | 1.22 [0.82 – 1.82] | 0.32 | 1.09 [0.67 – 1.77] | 0.72 |
| Any post-radiologic adverse event | Choledocholithiasis | 28.1 [19.3 – 36.9] | 33.1 [31.4 – 34.9] | 1.27 [0.82 – 1.98] | 0.29 | 1.47 [0.91 – 2.40] | 0.12 |
|  | Cholangitis | 59.7 [39.7 – 79.7] | 47.7 [44.7 – 50.7] | 0.62 [0.27 – 1.42] | 0.26 | 0.76 [0.29 – 2.01] | 0.58 |
| Any post-endoscopic adverse event | Choledocholithiasis | 14.1 [13.2 – 15.0] | 15.1 [14.9 – 15.4] | 1.09 [1.01 – 1.17] | 0.028 | 1.17 [1.07 – 1.28] | <0.001 |
|  | Cholangitis | 32.5 [27.9 – 37.1] | 33.8 [32.8 – 34.9] | 1.06 [0.86 – 1.32] | 0.57 | 1.01 [0.78 – 1.31] | 0.92 |
| **Any post-intervention mortality** | Choledocholithiasis | 0.5 [0.4 – 0.6] | 0.5 [0.5 – 0.5] | 1.00 [0.77 – 1.29] | 0.97 | 1.12 [0.82 – 1.53] | 0.48 |
|  | Cholangitis | 1.5 [0.5 – 2.5] | 1.5 [1.3 – 1.8] | 1.01 [0.51 – 1.99] | 0.98 | 1.91 [0.67 – 5.44] | 0.22 |
| Post-operative mortality | Choledocholithiasis | 0.4 [0.3 – 0.6] | 0.5 [0.4 – 0.5] | 1.05 [0.77 – 1.45] | 0.75 | 1.22 [0.83 – 1.79] | 0.31 |
|  | Cholangitis | 2.9 [0.1 – 5.7] | 1.9 [1.2 – 2.6] | 0.66 [0.22 – 1.94] | 0.45 | 3.64 [0.54 – 24.63] | 0.19 |
| Post-radiologic mortality | Choledocholithiasis | 4.3 [0.6 – 8.0] | 2.6 [2.0 – 3.2] | 0.60 [0.24 – 1.52] | 0.28 | 0.71 [0.25 – 2.02] | 0.52 |
|  | Cholangitis | 8.8 [0.0 – 20.2]* | 3.4 [2.4 – 4.5] | 0.37 [0.09 – 1.62] | 0.19 | 0.80 [0.09 – 6.96] | 0.84 |
| Post-endoscopic mortality | Choledocholithiasis | 0.5 [0.3 – 0.6] | 0.4 [0.4 – 0.5] | 0.87 [0.62 – 1.22] | 0.41 | 0.92 [0.63 – 1.35] | 0.66 |
|  | Cholangitis | 1.4 [0.4 – 2.4] | 1.4 [1.1 – 1.6] | 0.99 [0.46 – 2.14] | 0.98 | 1.95 [0.58 – 6.53] | 0.28 |

***** **Models were adjusted for age category, patient sex, race, insurance status, income quartile, Charlson comorbidity burden category, and weekend hospital admission.**

**^Δ^ Intervention refers to the use of any procedural intervention, including surgery/endoscopy/interventional radiology**

**Supplementary Figure 1: Annual trends in hospital discharges for choledocholithiasis with estimated annual percent change and joinpoint regression by age group.**


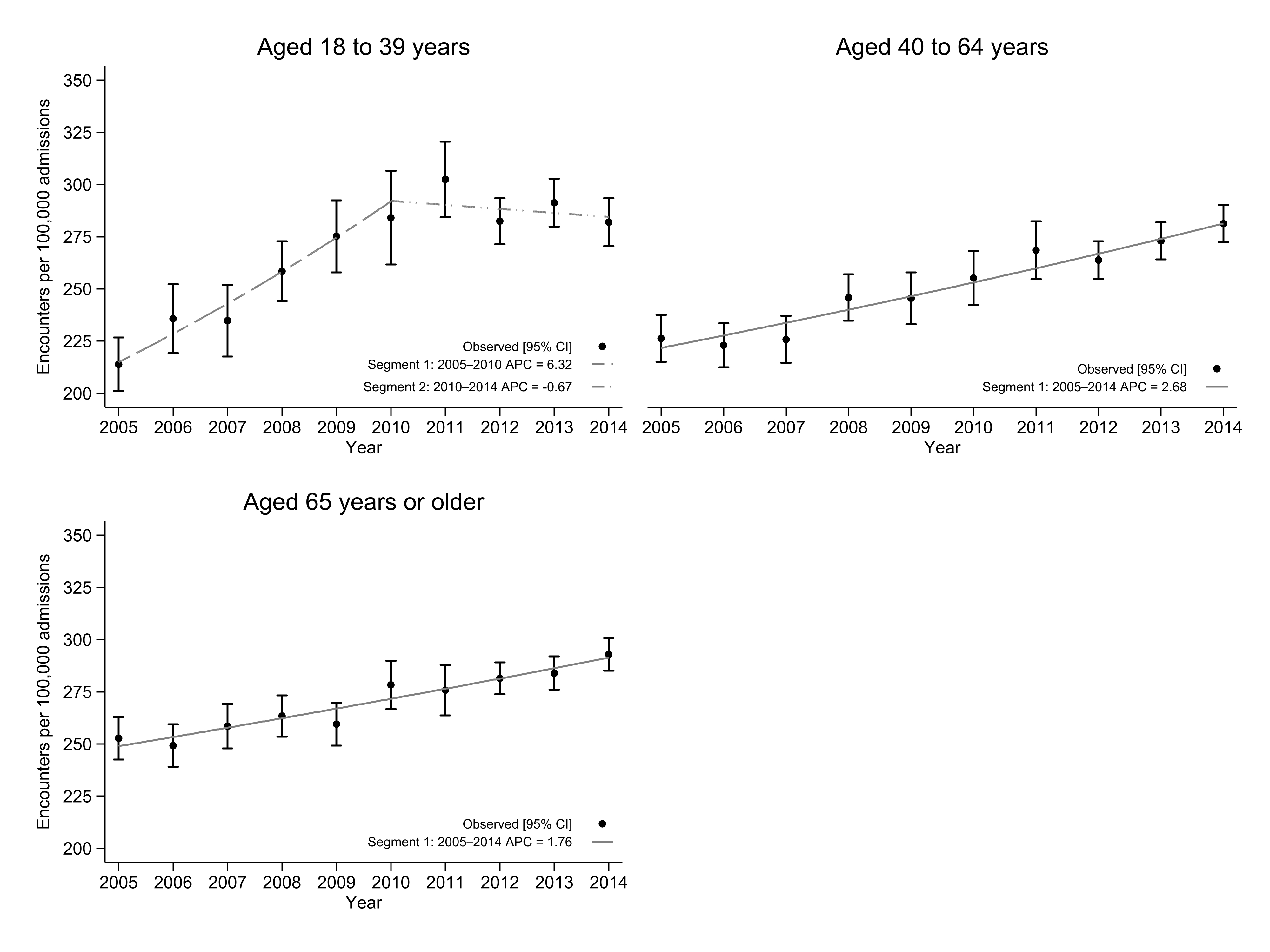


**Supplementary Figure 2: Estimated annual prevalence rates of choledocholithiasis and the sensitivity definition with presentation of cholangitis, overall.**


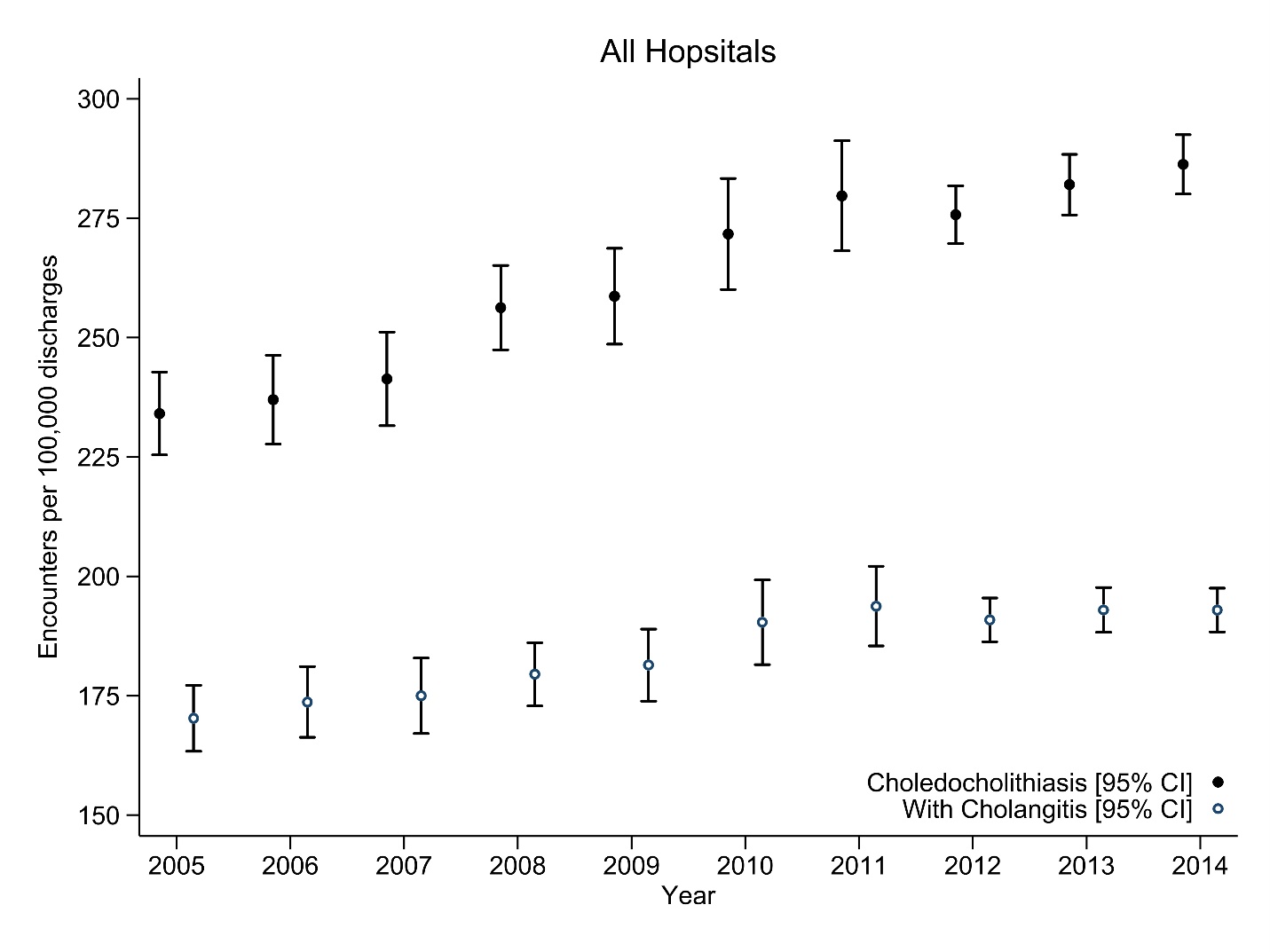


**Supplementary Figure 3: Annual trends in hospital discharges for cholangitis with estimated annual percent change and joinpoint regression by age group.**


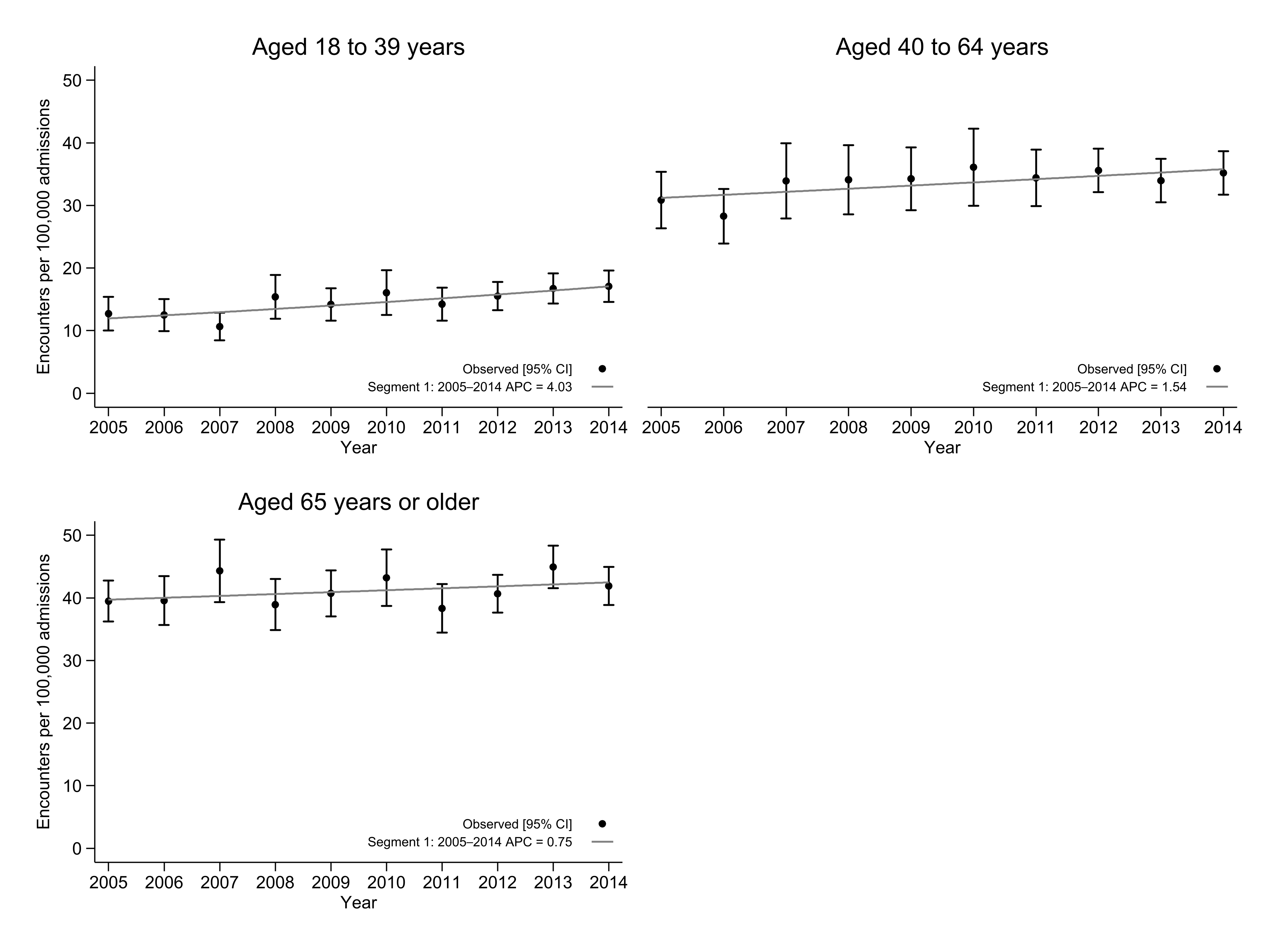

Supplement: Supplementary file 1 — Supplementary Material 1: Supplementary ICD-9 Coding, Tables, and Prevalance Trends [file 12876_2023_2868_MOESM1_ESM.docx]
